# Nanobody-based bispecific T-cell engager (Nb-BiTE): a new platform for enhanced T-cell immunotherapy

**DOI:** 10.1038/s41392-023-01523-3

**Published:** 2023-09-04

**Authors:** Xiao-mei Yang, Xuan-dong Lin, Wei Shi, Shen-xia Xie, Xia-ning Huang, Shi-hua Yin, Xiao-bing Jiang, Bruce D. Hammock, Zhi Ping Xu, Xiao-ling Lu

**Affiliations:** 1 Guangxi Key Laboratory of Nanobody Research, Guangxi Nanobody Engineering Research Center, College of Stomatology, Hospital of Stomatology, Laboratory Animal Center, School of Basic Medical Science, Pharmaceutical College, Guangxi Medical University, Nanning City, Guangxi 530021 P. R. China; 2 Department of Neurosurgery, Union Hospital, Tongji Medical College, Huazhong University of Science and Technology, Wuhan City, Hubei 430022 P. R. China; 3 Department of Entomology and Nematology, UCD Comprehensive Cancer Center, University of California, Davis, CA 95616 USA; 4 Australian Institute for Bioengineering and Nanotechnology, The University of Queensland, Brisbane, QLD 4072 Australia; 5 Institute of Biomedical Health Technology and Engineering and Institute of Systems and Physical Biology, Shenzhen Bay Laboratory, Shenzhen, 518107 P. R. China

**Keywords:** Drug development, Immunotherapy

**Dear Editor**,

A novel bispecific T-cell engager (BiTE) has been developed as an efficient immunotherapeutic molecule specifically bringing the T-cell and the tumor target together for enhanced immunotherapy. The general BiTE construct consists of two single-chain variable antibody fragments (scFvs) targeting a tumor-associated antigen (TAA) and a T-cell marker in tandem. The binding of BiTEs to tumor antigens induces immediate T-cells’ cytotoxicity against tumor cells without involving any typical costimulatory signals, specific TCR and MHC recognition. BiTE-based T-cell immunotherapies have succeeded in several preclinical and clinical trials, and one BiTE, i.e., Blinatumomab® (CD3×CD19), has been approved by FDA to treat leukemia. Even though there have been several new BiTEs developed to target various types of solid tumors, only Pasotuxizumab® (CD3×PSMA) has entered clinical trial for the treatment of prostate cancer.^1^ Treatment of solid tumors with these BITEs is far less satisfied with severe off-target adverse events, possibly due to (1) inherent mismatch of scFv molecules between their single light (VL)/heavy chain (VH) domain and hinge region causing low dual targeting, (2) off-target toxicity to non-tumoral cells, and (3) the complex tumor microenvironment as well as limited tumor penetration.^2,3^ To overcome these limitations of traditional BiTEs, developing new BiTEs is imperative, and this research has demonstrated that nanobody-based BiTE (Nb-BiTE) is one such candidate, with significantly increased therapeutic efficacy and reduced side effects in the animal model.

The variable domain of the heavy chain of heavy-chain-only antibodies, termed as nanobody (Nb), is considered the smallest unit with antibody functions found in nature, with many advantages over full-length antibody and scFv. Nbs have a much smaller molecular weight (14–18 kD) than full-length antibodies (~160 kD) and miniaturized antibody fragments (e.g., scFvs, ~35–40 kD), so they have deeper tissue penetration. Nbs are also featured with lower immunogenicity, higher specificity, stability, thermostability and solubility, and remarkably, ease of production.^4^ A humanized bivalent Nb against Von Willebrand factor developed by Ablynx, i.e., Caplacizumab (Cablivi™), is the first and the only therapeutic Nb approved by both EMA and FDA so far for the clinical treatment of adult thrombotic cytopenic purpura. The structural and functional advantages enable Nbs to be better alternatives to scFvs to construct BiTEs for efficient solid tumor treatments (Supplementary Fig. S1a),^4^ as demonstrated in a recent study. Here the ‘truncated’ BiTE construct was formed by linking an anti-PD-L1 Nb and an anti-CD3 scFv, which showed much higher efficacy in killing PD-L1-expressing tumor cells than that composed of two corresponding scFvs.^5^ As yet, there has been no report about BiTEs that are constructed with two Nbs for solid tumor immunotherapy.

In this letter, we report the development of a new BiTE (Nb-BiTE) platform that was exemplified with two Nbs, specific to the CD3 of T-cells and a TAA (e.g., CD105) for enhanced solid tumor treatment (Supplementary Fig. S1b). Different from other bispecific Nb fusions, Nb-BiTE more deliberately utilizes CD3 Nb to target CD3 (pan T-cell marker) of T-cells. As CD105 (also known as endoglin) has been preclinically and clinically well documented as a tumor target that is overexpressed in almost all solid tumor types as well as tumor-derived and tumor-associated vascular endothelial cells,^6^ this excellent TAA can be targeted with CD105 Nb in our proposed CD105-CD3/Nb-BiTE construct. We hypothesize that CD105-CD3/Nb-BiTE triggers configuration changes of the TCR/CD3 complex via the specific binding between CD3 Nb terminal and CD3 antigen of T-cell in combination with unique recognition and binding of TAA (i.e., CD105), thereby initiating membrane signal transduction and activating tumor-specific T-cells for proliferation and cytokine secretion. Hence, we particularly constructed CD3-targeting and CD105-targeting Nb-BiTE in the present study to prove our hypothesis. To our knowledge, there is no such a BiTE reported yet for the treatment of any type of solid tumors.

To screen CD3-specific Nb for CD105-CD3/Nb-BiTE construction, we first subcutaneously vaccinated a healthy camel with recombinant human CD3ε (hCD3) protein, and then collected and identified the purified CD3 Nb following our protocol for screening CD105-specific Nb.^6^ Six strains of 24 randomly selected colonies for CD3 Nb library had the typical Nb molecular weight (~14–18 kDa), and 4 showed specific binding to human T-cells (CD3^+^) but not 293T cells (CD3^-^) (Supplementary Fig. S2). Then, the gene sequences of the optimal CD3 Nb and our previously selected CD105 Nb^6^ were strung together via a flexible Gly_4_Ser linker to form CD105-CD3/Nb-BiTE for redirecting T-cells toward tumor cells and neovasculatural endothelial cells (Fig. 1a). TTE-CD3/Nb-BiTE was also produced similarly to provide nonspecific control where TTE was the chaotic gene sequence of CD105 Nb. The recombinant DNA of Nb-BiTE was sub-cloned into *p*ET-32a plasmid using PCR. The recombinant plasmid was then electroporated into BL21(DE3) *Escherichia coli* for prokaryotic expression of recombinant protein Nb-BiTE. The protein collection was then purified via chromatography, as confirmed by the single band at ~35 kD (Fig. 1b). This CD105-CD3/Nb-BiTE showed specific binding to both human peripheral blood-derived T-cells and CD105^+^ cells (Bel7404 and HepG2) but not CD105^-^ cells (SCC9 and 293T) (Fig. 1c). In contrast, the isotypic TTE-CD3/Nb-BiTE bound with neither CD105^+^ nor CD105^-^ cells (Supplementary Fig. S3). The specificity of CD105-CD3/Nb-BiTE was also validated by blocking assay of hCD105 or hCD3ε proteins (Supplementary Fig. S4).

**Fig. 1:**
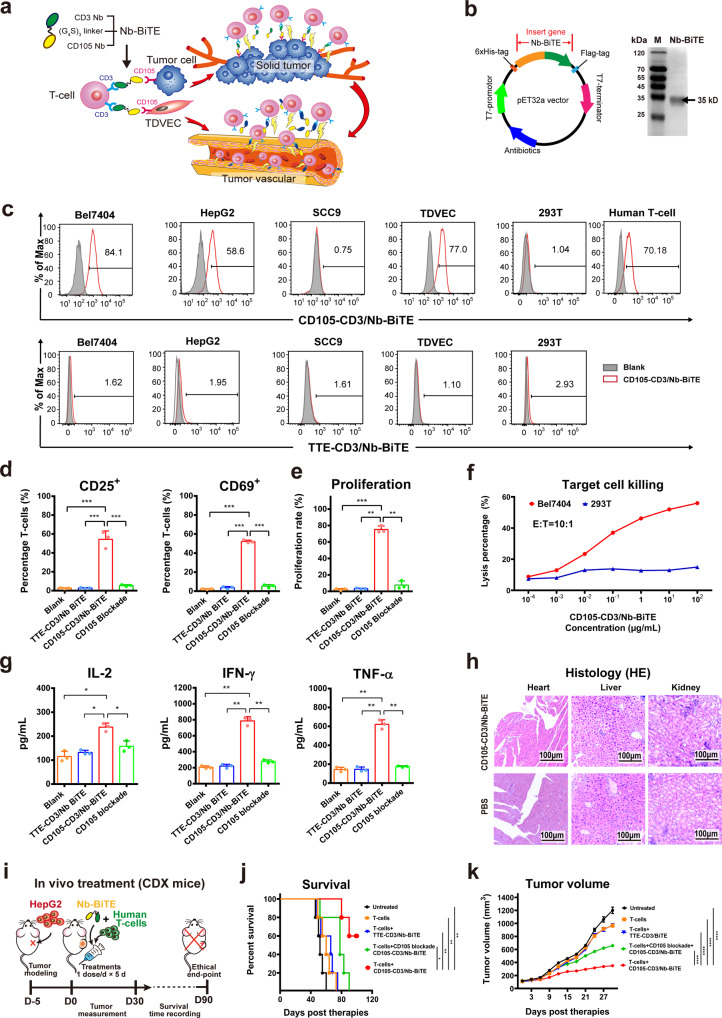
CD105-CD3/Nb-BiTE enhances the anti-tumor T-cell activities in vitro and in vivo, through specifically bridging and redirecting T-cells to CD105-expressing target cells. **a** The schematic illustration of the construction and the mechanism of CD105-CD3/Nb-BiTE. CD105-CD3/Nb-BiTE consists of two nanobodies (Nbs) specific to human CD3 of T-cells and CD105 of tumor cells connected with a triplicate Gly4Ser (Gly_4_Ser)_3_ flexible linker, and directs T-cells to target the CD105-overexpressing tumor cells and tumor vessels. **b** The schematic diagram of molecular structure of recombinant plasmid of Nb-BiTE (left panel) and the gel image of western blot assay for the imidazole-eluted CD105-CD3/Nb-BiTE fusion protein (~35 kDa molecular weight) (right panel). **c** Representative graphs of flow cytometry data showing binding specificity of CD105-CD3/Nb-BiTE to different cell types. An isotypic TTE-CD3/Nb-BiTE served as a nonspecific control. **d** CD105-CD3/Nb-BiTE increased the expression of CD25 and CD69 and activated T-cells, determined by PE-conjugated anti-hCD25 and –CD69 mAbs (eBioscience, USA). **e** CD105-CD3/Nb-BiTE promoted proliferation of human T-cells, determined by pre-staining of PKH26 dye (Sigma-Aldrich, USA). **f** CD105-CD3/Nb-BiTE enhanced specific killing ability of T-cells against CD105^high^ tumor cells (Bel7404) in a dose-dependent manner but not against CD105^-^ cells (293T). **g** CD105-CD3/Nb-BiTE increased the secretion of anti-tumor cytokines TNF-α, IFN-γ and IL-2 by T-cells under the stimulation of CD105^+^ tumor cells (HepG2), determined by commercial ELISA kits (R&D Systems, USA). **h** Representative H&E images of BALB/c mice receiving 10 μg of Nb-BiTE or PBS daily for 5 consecutive days (*n* = 3) showing no obvious histological difference between the two groups in the heart, and minimal histological differences in the liver and the kidney. Scale bar: 100 μm. **i** The schematic diagram shows CDX model in NOD/SCID mice and treatment scheme. CD105-CD3/Nb-BiTE (10 μg) and human T-cells (4 × 10^7^) were administered via tail-vein injection for 5 continuous days for each mouse to evaluate the anti-tumor efficacy. **j** The Kaplan–Meier survival curves of mice receiving treatments until the ethical endpoint (90 days). **k** The tumor volume profiles of mice receiving treatments in 30 days after the first administration. Statistics were performed using GraphPad Prism software. All data are represented as mean ± SEM of triplicates, from at least two independent experiments. The differences among groups were determined using the ANOVA analysis of variance test by LSD post hoc test. A two-tailed *p* value of <0.05 was considered statistically significant. Significance: ****p < 0.0001; ***p < 0.001; **p < 0.01; *p < 0.05

As-constructed CD105-CD3/Nb-BiTE remarkably activated primary T-cells, as evidenced by increased CD25^+^ and CD69^+^ subsets (Fig. 1d), and prominently facilitated their proliferation (Fig. 1e) in the presence of HepG2 target cells (CD105^+^). At the effector to target (E:T) ratio of 10:1, CD105-CD3/Nb-BiTE increased cytotoxicity of T-cells to CD105^+^ Bel7404 cells but not CD105^-^ 293T cells in a dose-dependent manner (Fig. 1f). Similarly, CD105-CD3/Nb-BiTE showed a much higher lysis rate of CD105^+^ targets than TTE-CD3/Nb-BiTE, which was blocked specifically by hCD105 protein, suggesting the high CD105 specificity of CD105-CD3/Nb-BiTE (Supplementary Fig. S5). In consistence, CD105-CD3/Nb-BiTE significantly elevated secretion of major anti-tumor cytokines (IL-2, IFN-γ and TNF-α) by T-cells (Fig. 1g). These results demonstrated the high potency of CD105-CD3/Nb-BiTE in enhancing the anti-tumor activity of T-cells.

Furthermore, CD105-CD3/Nb-BiTE showed excellent safety and therapeutic efficacy in vivo. Since systemic bioavailability is considered a basic prerequisite for a therapeutic product, we systemically injected 10 μg of CD105-CD3/Nb-BiTE into healthy BALB/c mice daily for 5 consecutive days. No significant pathological change or inflammatory cell enrichment in tested organs was found, suggesting CD105-CD3/Nb-BiTE is histotoxicity free (Fig. 1h). We particularly established a cancer cell line-derived xenograft (CDX) model in NOD/SCID mice by subcutaneously implanting HepG2 cells, following the treatment with Nb-BiTE and human T-cells (Fig. 1i). Clearly, the mice receiving CD105-CD3/Nb-BiTE and T-cells showed much longer survival than all other controls, with 60% mice being still alive at the test endpoint (Fig. 1j). This Nb-BiTE also significantly reduced the tumor size, with the tumor growth being well inhibited (Fig. 1k) and the body weight of treated groups seemed to be relatively stable in the first month (Supplementary Fig. S6). The improved mental and physical conditions of mice were observed as well. Moreover, CD105-CD3/Nb-BiTE treatment also significantly increased intratumoral infiltration of imported T-cells (Supplementary Fig. S7). These data collectively demonstrate that CD105-CD3/Nb-BiTE induced targeted anti-tumor potency of T-cells, and the underlying mechanism warrants further investigation.

In summary, the novel CD105-CD3/Nb-BiTE showed a high potential for solid tumor immunotherapy. This landmark study may demonstrate that this new Nb-BiTE construct is a universal platform that potently promotes T-cell immunotherapy, attributed to its own comparative advantages, such as small size, structural simplicity, stability, versatility, ease of design and production, and most importantly, high specificity and safety. Such a CD3 Nb-cored Nb-BiTE platform has been actively developed for distinct TAA targets currently in our lab (by altering the CD105 Nb with other Nb specific to alternative target), promising immunotherapy for solid tumor treatment.

### Supplementary information

Supplementary Materials for Nanobody-based bispecific T-cell engager (Nb-BiTE): a new platform for enhanced T-cell immunotherapy

## Data Availability

Data in this study are included in the main article or in supplementary materials.
