# Supplementary material for: Nanobody-based bispecific T-cell engager (Nb-BiTE): a new platform for enhanced T-cell immunotherapy
Source: Signal Transduct Target Ther. 2023 Sep 4;8:328. doi: 10.1038/s41392-023-01523-3 (PMC10475457; doi:10.1038/s41392-023-01523-3)
Supplement: Supplementary file 1 — Supplementary Materials for Nanobody-based bispecific T-cell engager (Nb-BiTE): a new platform for enhanced T-cell immunotherapy [file 41392_2023_1523_MOESM1_ESM.docx]

**Supplementary Materials for**

**Nanobody-based** **bispeciﬁc T-cell engager (Nb-BiTE): a new platform for enhanced T-cell immunotherapy**

Xiao-mei Yang^*^, Xuan-dong Lin^*^, Wei Shi^*^, Shen-xia Xie, Xia-ning Huang, Shi-hua Yin, Bruce D. Hammock, Xiao-bing Jiang, Zhi Ping Xu ^✉️^, Xiao-ling Lu ^✉️^

^*^ These authors contributed equally to this work

^✉️^Correspondence to: [luxiaoling@gxmu.edu.cn](mailto:luxiaoling@gxmu.edu.cn) or [gordonxu@uq.edu.au](mailto:gordonxu@uq.edu.au)

**This PDF file includes:**

Figures S1 to S7


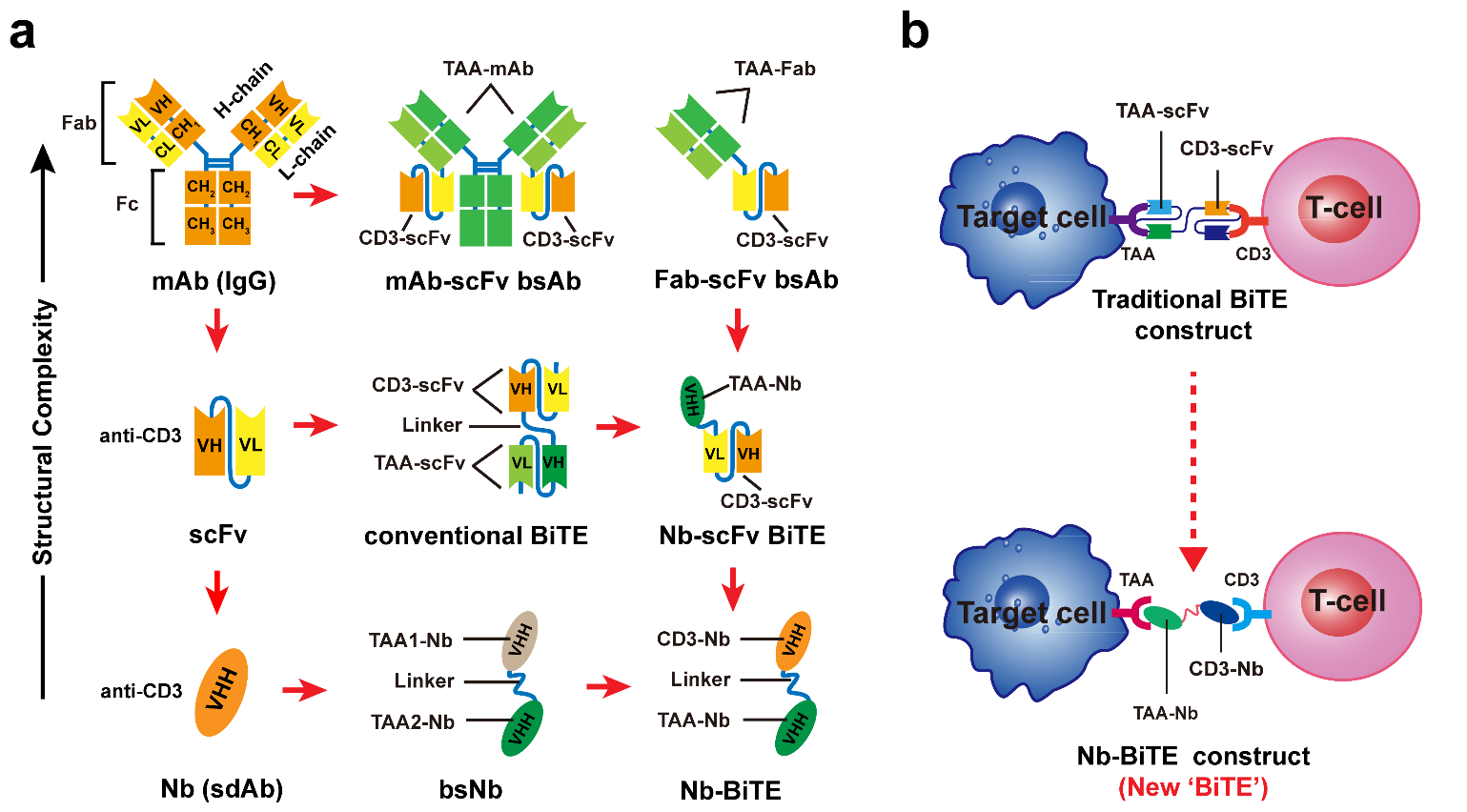


**Fig. S1. Comparison of the molecular structure of different antibody types for understanding the characteristics of the new Nb-BiTE construct. a** Schematic representation of conventional mAb, scFv, Nb and their derivates. The CD3 Nb-cored Nb-BiTE construct as a new potent tool for solid tumor immunotherapies has reduced molecular size and complexity than any of full-length mAb, mAb-scFv bsAb ^1^, Fab-scFv bsAb ^2^, BiTEand Nb-scFv BiTE^2^ (also termed “LiTE”) ^3^, and has specific targeting to pan T-cells rather than the other bsNb whom shares similar structure and crytal size. Arrows show the evolutionary relationships between indicated antibody types ^4-7^. **b** Schematic representation of comparison between traditional BiTE format and the Nb-BiTE format (the new ‘BiTE’).

**
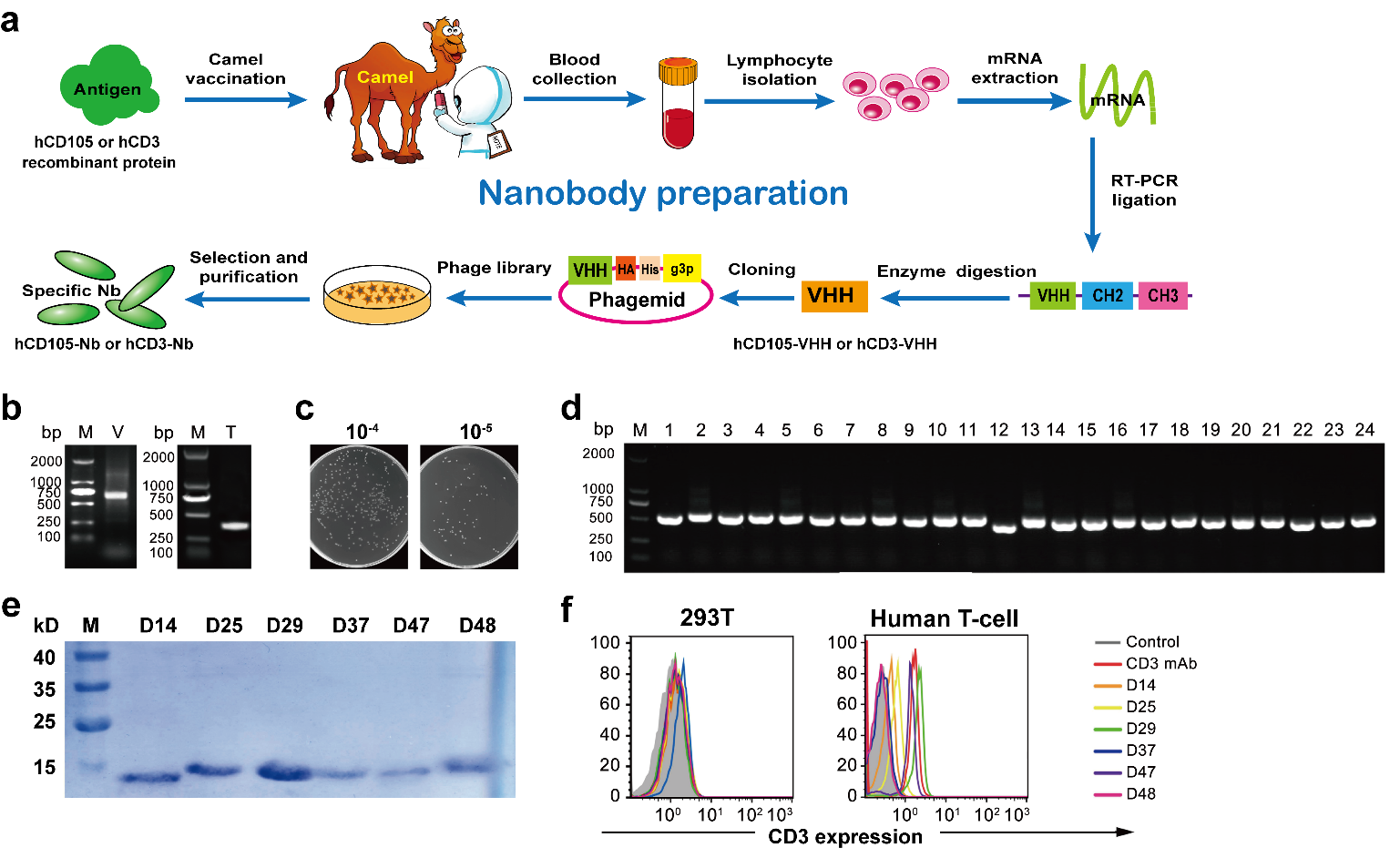
**

**Fig. S2. Preparation of nanobodies. a** Schematic flow of general procedure for the preparation of specific nanobodies (Nbs) targeting human CD3 (hCD3) or human CD105 (hCD105). **b** Agarose gel electrophoresis (AGE) illustrate the bands of CD3-specific VHH DNA fragments obtained by two-step PCR. **c** The library size was measured by counting the number of colonies after serial dilution. **d** AGE demonstrates the PCR amplification products for determination of the insertion of CD3-specific VHH genes from 24 randomly selected colonies. **e** Sodium dodecyl sulfate polyacrylamide gel electrophoresis (SDS-PAGE) analysis of purified CD3 Nb strains. **f** Representative graph for flow cytometry analyses of six CD3 Nb strains that strongly bind to the CD3-positive T-cells but not CD3-negative 293T cells.


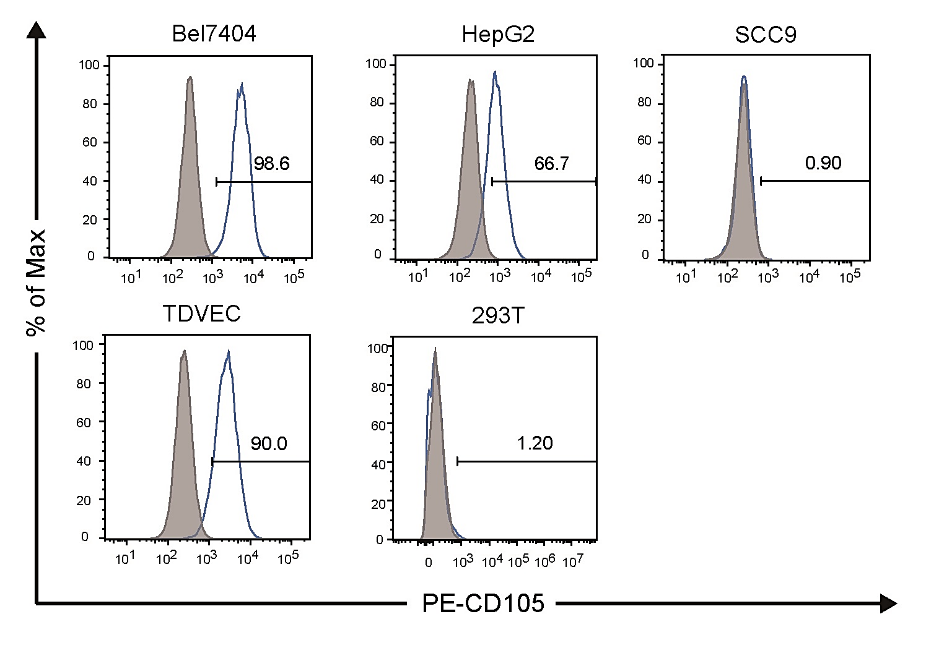


**Fig. S3. Expression of CD105 in various human-sourced cell lines.** Representative graph for flow cytometric analyses show the percentage of CD105-positive cells. A PE-conjugated human CD105 (Endoglin) mAb (SN6; eBioscience, USA) was used in this study.


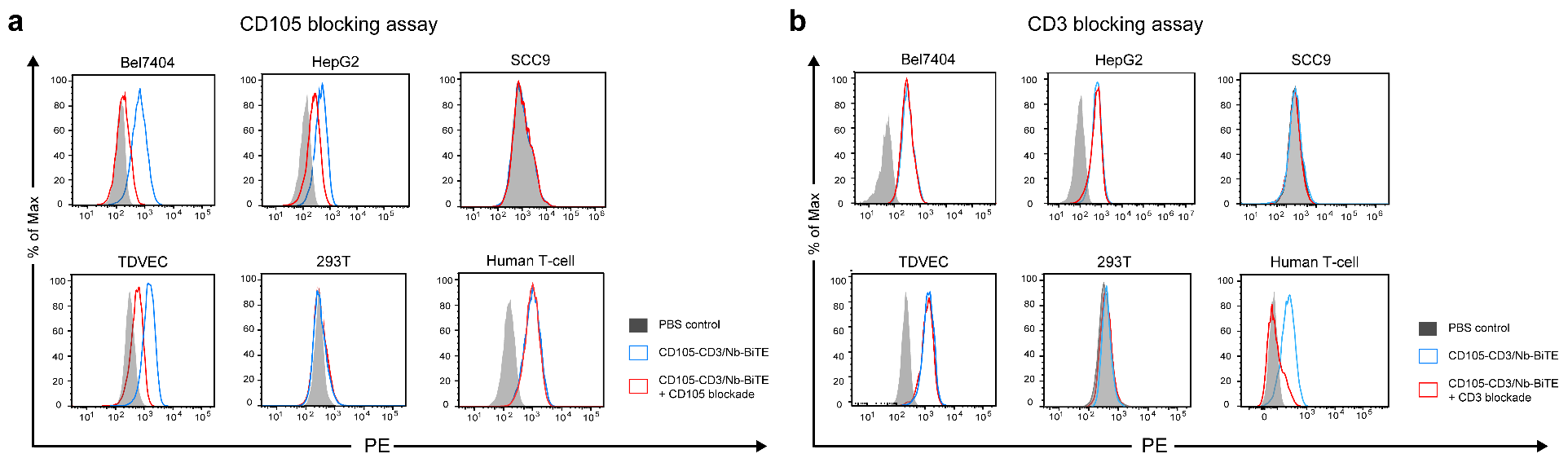


**Fig. S4. CD105-CD3/Nb-BiTE specifically binds CD105-expressing target cells and CD3-expressing human T-cells**. To verify the cellular binding specificity of CD105-CD3/Nb-BiTE, soluble recombinant hCD105 protein and hCD3ε protein were respectively added as competitors respectively for the CD105 and CD3 epitopes on target cells. CD105-expressing human cells (Bel7404, HepG2 and TDVEC), CD105-negative cells (SCC9 and 293T) or human primary T-cells were incubated with CD105-CD3/Nb-BiTE alone or the mixture of CD105-CD3/Nb-BiTE and equivalent moles of recombinant hCD105 protein (CD105 blockade) **(a)** or hCD3ε protein (CD3 blockade) **(b)** diluted in PBS, or PBS vehicle. PE-conjugated anti-His mAb (Sigma-Aldrich, USA) was then used to stain the CD105-CD3/Nb-BiTE bounded on the cell’s surface, followed with flow cytometry analysis of binding rate to assess the effects of CD105 blockade and CD3 blockade (representative graph shown as indicated).


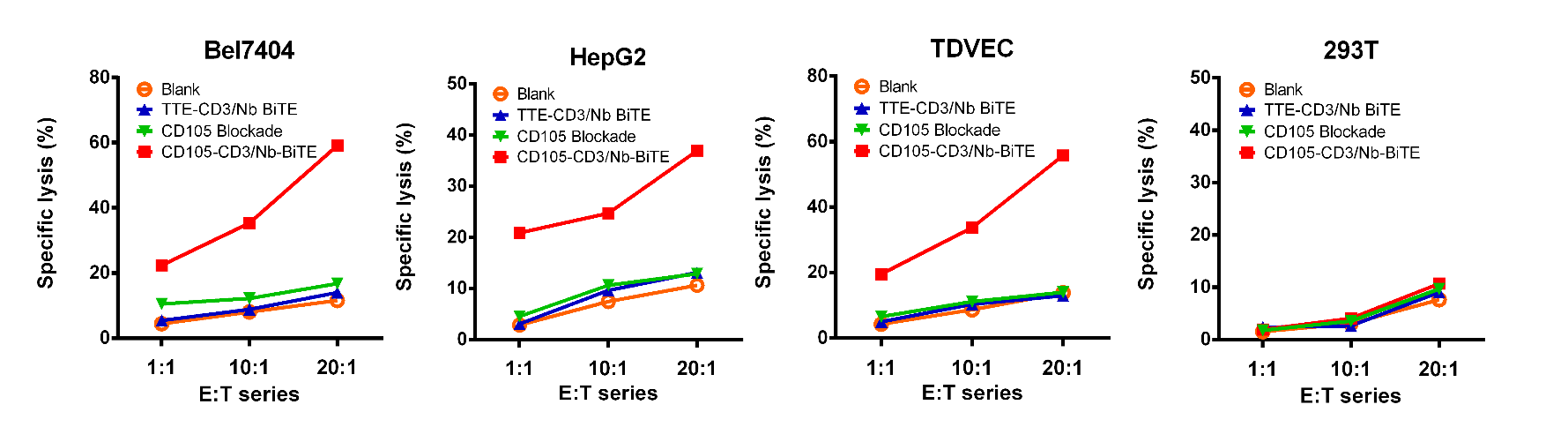


**Fig. S5. CD105-CD3/Nb-BiTE mediates and enhanced CD105-specific T-cell responses against multiple tumor cell lines or tumor-associated cells.** Flow cytometry analyses showed that CD105-CD3/Nb-BiTE specifically enhanced the killing ability of human T-cells against CD105^+^ tumor cells (HepG2 and Bel7404) and tumor-derived vein endothelial cells (TDVECs) depended on E:T ratio, but not CD105^-^ (293T) cells, *in vitro*, shown as curves of E:T ratio versus target cell lysis rate. Recombinant hCD105 protein was used as a blocker to determine the CD105-targetability. The isotypic TTE-CD3/Nb-BiTE served as the non-specific control for CD105-CD3/Nb-BiTE.





**Fig. S6. Body weight changes of tumor-bearing mice receiving CD105-CD3/Nb-BiTE and T-cells treatment.** The mice were weighted every 3-days after first dose of treatment (Day 0) until Day 30. Significant weight loss was observed only in the untreated group. Statistics was performed using a GraphPad Prism software. Data are represented as mean ± SEM of triplicates, from at least two independent experiments. The differences among groups were determined using the ANOVA analysis of variance test by LSD post-hoc test. A two-tailed *p*-value of < 0.05 was considered statistically significant. Significance: ns: *p* >0.05; **: *p* < 0.01.





**Fig. S7. CD105-CD3/Nb-BiTE increases the infiltration of T-cells into tumor tissue.** Subcutaneous tumors were separated after the in vivo treatment trial, minced and homogenized into single-cell suspensions. Cells were washed with sterile PBS and collected. A PE-conjugated hCD3 mAb (OKT3; eBioscience, USA) was used to stain the cells. Flow cytometry analyses showed that CD105-CD3/Nb-BiTE specifically increased the percentage of human T-cells (CD3^+^ population) in the total cells within the tumors, in comparison of isotypic TTE-CD3/Nb-BiTE or any else control group, as indicated. Statistics was performed using a GraphPad Prism software. Data are represented as mean ± SEM of triplicates, from at least two independent experiments. The differences among groups were determined using the ANOVA analysis of variance test by LSD post-hoc test. A two-tailed *p*-value of < 0.05 was considered statistically significant. Significance: ****: *p* < 0.0001; ***: *p* < 0.001; **: *p* < 0.01.

**References**

1. Wu, Z. et al. Development of a Tetravalent Anti-GPA33/Anti-CD3 Bispecific Antibody for Colorectal Cancers. *Mol. Cancer Ther.* **17**, 2164-2175 (2018).
2. Chen, J. et al. A Novel Bispecific Antibody Targeting CD3 and Lewis Y with Potent Therapeutic Efficacy against Gastric Cancer. *Biomedicines* **9**, 1059 (2021).
3. Mølgaard, K. et al*.* Bispecific light T-cell engagers for gene-based immunotherapy of epidermal growth factor receptor (EGFR)-positive malignancies. *Cancer Immunol. Immunother.* **67**, 1251-1260 (2018).
4. Liu, Y. et al. Development of a Bispecific Nanobody Targeting CD20 on B-Cell Lymphoma Cells and CD3 on T Cells. *Vaccines* **10**, 1335 (2022).
5. Ma, L. et al. A novel bispecific nanobody with PD-L1/TIGIT dual immune checkpoint blockade. *Biochem. Biophys. Res. Commun.* **531**, 144-151 (2020).
6. de Bruin, R. C. G. et al. A bispecific nanobody approach to leverage the potent and widely applicable tumor cytolytic capacity of Vγ9Vδ2-T cells. *Oncoimmunology* **7**, e1375641 (2017).
7. Kovalchuk, B. et al. Nintedanib and a bi-specific anti-VEGF/Ang2 nanobody selectively prevent brain metastases of lung adenocarcinoma cells. *Clin. Exp. Metastasis* **37**, 637-648 (2020).
